# Molecular biology of the blood-brain and the blood-cerebrospinal fluid barriers: similarities and differences

**DOI:** 10.1186/2045-8118-8-3

**Published:** 2011-01-18

**Authors:** Zoran Redzic

**Affiliations:** 1 Department of Physiology, Faculty of Medicine, Kuwait University, SAFAT 13110, Kuwait

## Abstract

Efficient processing of information by the central nervous system (CNS) represents an important evolutionary advantage. Thus, homeostatic mechanisms have developed that provide appropriate circumstances for neuronal signaling, including a highly controlled and stable microenvironment. To provide such a *milieu *for neurons, extracellular fluids of the CNS are separated from the changeable environment of blood at three major interfaces: at the brain capillaries by the blood-brain barrier (BBB), which is localized at the level of the endothelial cells and separates brain interstitial fluid (ISF) from blood; at the epithelial layer of four choroid plexuses, the blood-cerebrospinal fluid (CSF) barrier (BCSFB), which separates CSF from the CP ISF, and at the arachnoid barrier. The two barriers that represent the largest interface between blood and brain extracellular fluids, the BBB and the BCSFB, prevent the free paracellular diffusion of polar molecules by complex morphological features, including tight junctions (TJs) that interconnect the endothelial and epithelial cells, respectively. The first part of this review focuses on the molecular biology of TJs and adherens junctions in the brain capillary endothelial cells and in the CP epithelial cells. However, normal function of the CNS depends on a constant supply of essential molecules, like glucose and amino acids from the blood, exchange of electrolytes between brain extracellular fluids and blood, as well as on efficient removal of metabolic waste products and excess neurotransmitters from the brain ISF. Therefore, a number of specific transport proteins are expressed in brain capillary endothelial cells and CP epithelial cells that provide transport of nutrients and ions into the CNS and removal of waste products and ions from the CSF. The second part of this review concentrates on the molecular biology of various solute carrier (SLC) transport proteins at those two barriers and underlines differences in their expression between the two barriers. Also, many blood-borne molecules and xenobiotics can diffuse into brain ISF and then into neuronal membranes due to their physicochemical properties. Entry of these compounds could be detrimental for neural transmission and signalling. Thus, BBB and BCSFB express transport proteins that actively restrict entry of lipophilic and amphipathic substances from blood and/or remove those molecules from the brain extracellular fluids. The third part of this review concentrates on the molecular biology of ATP-binding cassette (ABC)-transporters and those SLC transporters that are involved in efflux transport of xenobiotics, their expression at the BBB and BCSFB and differences in expression in the two major blood-brain interfaces. In addition, transport and diffusion of ions by the BBB and CP epithelium are involved in the formation of fluid, the ISF and CSF, respectively, so the last part of this review discusses molecular biology of ion transporters/exchangers and ion channels in the brain endothelial and CP epithelial cells.

## Introduction

A constant and well-controlled composition of the extracellular fluid in the central nervous system (CNS) is essential for efficient neuronal processing. Invertebrate nervous systems, which are far less complex than the mammalian brain, are protected from fluctuations in composition of body fluids by a barrier that is formed by glial cells and this arrangement also applies to some ancestral vertebrates. With the CNS becoming more complex during evolution, an endothelial barrier appeared, giving a strong selective advantage. Consequently, all existing vertebrates, except for a few fish species, have endothelial blood-brain barriers (BBB).

The BBB and the blood-cerebrospinal fluid barrier (BCSFB) are formed by brain endothelial cells (BECs) and choroid plexus (CP) epithelial cells, respectively. The BBB and the BCSFB are not only anatomical barriers, but also dynamic tissues that express multiple transporters, receptors and enzymes. Brain capillaries are closely associated with perivascular astrocytic end-feet, pericytes and microglia that influence BBB permeability and, together with brain endothelial cells, constitute a "neurovascular unit".

The two main functions of these barriers are to impede free diffusion between brain fluids and blood and to provide transport processes for essential nutrients, ions and metabolic waste products. Hence, the aim of this review is to address similarities and differences in the molecular biology of cellular junctions, solute carrier transporters, ATP-binding cassette transporters and ion transporters at the BBB and the BCSFB.

## Morphology of the BBB and BCSFB

Although there are several similar features between the blood-brain barrier (BBB) and the blood-cerebrospinal fluid barrier (BCSFB), it should be kept in mind that the cellular basis of these two structures as well as their primary functions differ: BBB is located in brain capillaries and, thus, it is an endothelial structure with its main role to protect the brain from physiological fluctuations in plasma concentrations of various solutes and from blood-borne substances that could interfere with neurotransmission, but at the same time to provide mechanisms for exchange of nutrients, metabolic waste products, signaling molecules and ions between the blood and the brain ISF. In contrast to this, the BCSFB is created by a layer of a modified cuboidal epithelium, the CP, that secretes cerebrospinal fluid (CSF) and this process could be considered as main function of this epithelium. The differences in principal function are related to differences in morphology and molecular biology.

Brain capillaries express complex morphology that provide the restrictive characteristics of the endothelial layer with regard to diffusion of solutes; this is an essential feature to protect the brain from unwanted solutes from blood with tight junctions (Tjs) that interconnect adjacent endothelial cells and occlude the paracellular spaces. In addition, BECs show low pinocytotic activity and the endothelium is further secluded by a layer of astrocytic end feet and pericytes on the brain side that place additional restrictions on permeability. Thus, the BBB *in vivo *provides high resistance to movement of ions, with transendothelial electrical resistance (TEER) being in the range of 1500 Ω cm^2 ^(pial vessels), which is quite high when compared to TEER of 3-33 Ω cm^2 ^in other tissues [1]. The total capillary surface area in the brain is about 100-150 cm^2 ^g^-1 ^[2], which when estimated for the whole brain approximates 20 m^2 ^[3], suggesting that the BBB can be considered as a large and thin membrane, providing ideal conditions for exchange processes between blood and brain interstitial fluid (ISF). When considering the total area available for exchange, it should be noted that brain capillaries are perfused all the time, but they shift to high blood flow with an increase in cerebral blood flow (CBF), or to low blood flow with a decrease in CBF [4].

**Figure 1:**
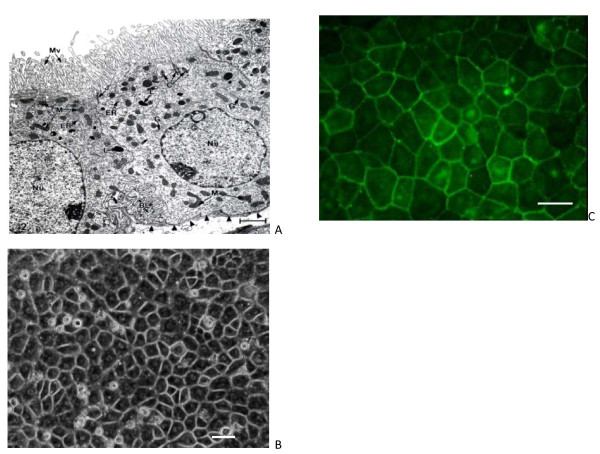
**Morphology of choroid plexus epithelium (CPE) *in situ *and in primary culture**. A. Ultrastructure: CP from lateral ventricle of an adult Sprague-Dawley rat. Apical membrane (CSF-facing) shows numerous microvilli (Mv) and many intracellular mitochondria (M). J refers to the tight junction welding two cells at their apical poles. C: centriole. G and ER: Golgi apparatus and endoplasmic reticulum. Nucleus (Nu) is oval and has a nucleolus. Arrowheads point to basal lamina at the plasma face of the epithelial cell; the basal lamina separates the CPE above from the interstitial fluid below. Basal labyrinth (BL) is the intertwining of basolateral membranes of adjacent cells. Choroidal morphology resembles proximal tubule, consistent with both cell types rapidly turning over fluid. Scale bar = 2 μm, reproduced from [248] with permission. B. Phase-contrast micrographs of 8d-old sheep CPE cells cultured on laminin-coated filters shows a typical cobblestone arrangement of polygonal cells (scale bar 20 μm). C. Eight-day-old sheep CPE cells grown on laminin-coated filters were stained with primary antibodies against occludin and then with FITC conjugated secondary antibodies. A continuous circumferential distribution of fluorescence consistent with the establishment of TJs in CPEC monolayer is shown. Scale bar 20 μm. Images B and C reproduced from [257].

Choroid plexuses are villous structures floating in the CSF and attached to the ventricular ependyma by a stalk. The ependyma is continuous with the epithelial layer of the CP which is composed of a single layer of cells filled with mitochondria and joined together by tight TJs (Figure 1) [5]. The TEER offered by these TJs cannot be measured *in vivo *in most animals. However, *in vitro *measurements using the single-sided fourth ventricle CP of the bull frog maintained in an Ussing chamber suggested values of about 150 Ω cm^2 ^[6], much less than the resistance of the BBB. The low value of TEER would suggest that the CPs fall into the class of leaky epithelia, similar to some segments of the kidney and gut, which form an isotonic fluid and do not generate steep transepithelial concentration gradients across the tissues [7]. These leaky epithelia can secrete large volumes of fluid but use relatively little energy for this process. CP epithelial cells posses a dense apical coat of microvilli, while kinocilia are rarely found; in contrast to this, the apical surface of ventricular ependymal cells demonstrates a large number of kinocilia [8], with rare microvilli of variable size. Between the lateral walls of the CP epithelial cells are complex interdigitations particularly apparent close to the blood side of the tissue laying on a basal lamina that demarcate the inner stroma of a highly vascularized connective tissue; these interdigitations expand the surface area of the CP [9].

## Molecular biology of cell junctions at the BBB and BCSFB

Brain endothelial cells (BECs) and CP epithelial (CPE) cells are connected at a junctional complex by the TJ and adherens junctions (AJ) [10]. BECs also express gap junctions but their functional significance is not clear. All TJ and AJ are composed of transmembrane proteins and cytoplasmic plaque proteins; plaque proteins cluster integral TJ proteins and form a platform for interaction with scaffolding and signaling proteins. In addition, a circumferential actin belt that encircles each endothelial/epithelial cell at the level of TJs is important for formation and normal function of TJs.

### Protein structure of tight junctions

**Figure 2:**
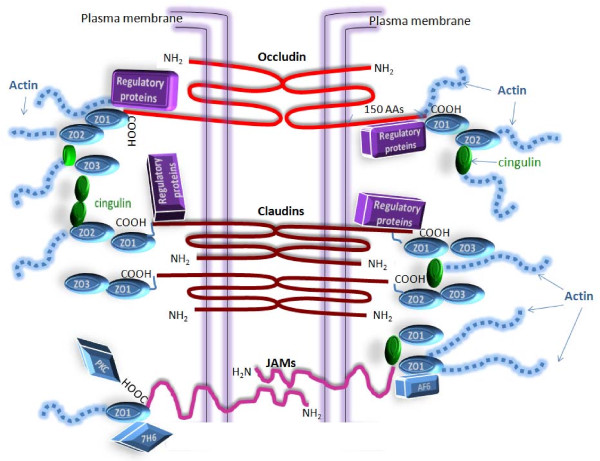
**Schematic representation of tight junctions between two adjacent cells**. In general, TJs at the BBB and in the CP epithelium are similar, but they express different claudins (that are not shown in this figure). This is probably an important structural difference underlying the lower values of TEER across CP epithelium compared to TEER values across the brain endothelium.

Transmembrane proteins of the TJ include occludin, claudins and junctional adhesion molecules (JAM)-A, B and C [11,12] (Figure 2). Occludin structure appears to be essential for normal occluding function of TJs in both BBB and BCSFB. Occludin possesses two extracellular loops, four trans-membrane domains and three cytoplasmic domains; the cytoplasmic domains include one intracellular short turn, N-terminal domain and a 150 amino-acids long carboxyl (C-) -terminal domain [12,13] (Figure 2). Extracellular loops provide the gate-like structure of TJs; it is believed that second loop mainly determines the TEER [14]. The C-terminal domain associates with zonulla occludens proteins (ZO) -1, ZO-2 and ZO-3 and interacts with regulatory proteins, such as protein kinase C, tyrosine kinase and phosphoinositide 3-kinase [12,15]. Both occludin and claudins are phospho-proteins that change conformation upon phosphorylation/dephosphorylation of the side chain hydroxyl group, which affects interaction with other proteins; therefore, regulatory proteins mainly posses kinase or phosphatase activities. Dephosphorylation of occludin causes disassembly of its association with ZO proteins. Deletion of occludin in mice results in postnatal growth retardation, although the TJs themselves appear to function normally [16], which suggests that other TJ proteins compensate for the lack of occludin. Occludin deletion from embryonic stem cells did not prevent differentiation of these cells into polarized epithelial cells with clear TJs [17]. The N-terminal part of occludin has an important role in a TJ assembly; this activity was revealed by an experiment in which abnormal occludin that lacks N-terminal domain caused a damaging effect on the TJ function of endothelial cell monolayers *in vitro*. Those monolayers failed to develop high TEER and developed increased paracellular diffusion of small polar molecules [18]. Occludin is also subject to endocytic recycling with two proteins associated to TJs, a member of the Rab family G-proteins, Rab13, and a Rab13-binding protein, MICAL-L2 (molecule interacting with CasL-like 2) mediating the specific endocytic recycling of occludin (but not other membrane proteins, like transferrin receptor), which is important for maintenance of functional TJs [19]. A study has revealed that in Alzheimer's disease (AD) and in vascular dementia there were significantly more occludin-positive astrocytes and oligodendrocytes in the frontal white matter than in age-matched controls [20], which may indicate autophagy of TJ proteins by the surrounding glial cells.

Claudins are the principal barrier-forming proteins, which include a multigene family consisting of at least 24 members in mammals and are an essential structural component of TJ strands. All claudins show the same structural pattern: four membrane-spanning regions, two extracellular loops and two cytoplasmic domains, a short N-terminal sequence and a long C-terminal sequence [21] (Figure 2). Two neighboring claudins from two adjacent cells form TJ strands through homophilic claudin-claudin interactions [22]. Extracellular loops determine paracellular charge selectivity, so each type of claudin regulates the diffusion of a group of molecules of a certain size. Deletion of claudin 5 in mice showed detrimental effects on the brain causing early death; those effects were due to a size-selective loosening of the BBB for molecules with MW<800 Da [23]. The claudin C-terminus binds cytoplasmic proteins, particularly ZO-1, ZO-2, and ZO-3 [24] (Figure 2). Proper interaction of claudins is essential to selectively limit paracellular ion movement, an action which produces the high TEER of the BBB. It appears that the differences in claudin content between the two barriers play an important role in the observed differences in TEERs between the BBB and the BCSFB [25,26]: claudins 3, 5, 12 and probably 1 are present at the BBB [10,23], while claudin 1, 2, 3 and 11 are expressed in the CP epithelium [27]. It was initially believed that claudin-1 was the most abundant TJ protein in the CPE and a marker of CP TJs [27]; this was later realized to be an artifact due to a cross-reaction of the anti-claudin-1 antibodies with claudin-3; it appears that claudin-3 is the most abundant claudin in CPE [28].

It was known from Goldman's second experiment [29] that the lining of the ventricular walls, which consists of a layer of ependymal cells, does not restrict diffusion of solutes. The molecular basis for this is that ependymal cells in mammals do not express TJs [30]. However, complex TJs were described in some fish and amphibia [31,32]. Also, it was reported in mammalian brain during embryonic development that the ependymal cell layer forms cellular junctions similar to TJs and provides this layer with barrier properties [33]. This might indicate that disappearance of this CSF-brain barrier could be related to the development of the more effective epithelial BCSFB in adult mammals.

JAMs A,-B and C are members of the immunoglobulin superfamily that have a membrane-spanning domain, an extracellular domain, an extracellular N-terminus, and a cytoplasmic C-terminus [34] (Figure 2). JAMs are expressed at the intracellular junctions of BECs and CPEs and have different patterns of homophilic and heterophilic interactions with JAM molecules on the adjacent cell, forming dimers that are part of the tight junction structure [35]. The short C terminal tail contains a domain which mediates interactions with ZO-1, cingulin, junction-associated protein AF6, tight-junction-associated protein antigen 7H6 and scaffold proteins [36] and also includes phosphorylation sites that may serve as substrates for protein kinase C (PKC) [37]. It is believed that JAMs are involved in the localization of ZO-1 and occludin in TJ complexes [34]. Transmembrane TJ proteins are linked to the cytoskeleton by scaffolding ZO proteins 1, 2 and 3 in BECs and in CPE [10,30]. These proteins belong to a family of membrane-associated guanylate kinase proteins. ZO proteins provide the cytoskeletal anchorage for the TJ proteins and are also involved in control of spatial distribution of claudins. Cingulin is a myosin-like protein that binds preferentially to ZO proteins at the globular head, to other cingulin molecules at the tail and to actin. Actin has known binding sites on all of the ZO proteins, on claudin, occludin and cingulin [38].

A study on rat BBB that used serial analysis of gene expression (SAGE) provided a comprehensive gene expression profile of rat BECs from freshly-collected brain microvasculature and has revealed that the SAGE tag for claudin 5 was 16^th ^of the 50 most abundant tags enriched in BECs [39] with a relative abundance in rat BEC SAGE catalog 52 tags/100.000. Other TJ protein transcripts were less abundant: claudin 11 (18/100.000), ZO-2 (11/100.000) and ZO-1 (3/100.000) and they were not among the 50 most abundant tags enriched in BECs [39].

Through interactions with other proteins and/or as a consequence of cell signaling, TJs in the brain are dynamic structures; spatial distributions of proteins can be changed under various circumstances. Effects of signaling on TJ expression and integrity have been studied for pathophysiological conditions, including cerebral ischemia *in vivo*, conditions that mimic ischemia *in vitro *and inflammation. Claudin 5 expression was reduced and localization in BECs altered by hypoxia *in vitro*; changes were accompanied by a decrease in TEER [40]. A decrease in occludin and ZO-1 expression in BECs after cerebral embolism has been reported [41] and localization of occludin, ZO-1, and ZO-2 proteins was altered after hypoxia *in vitro *[42]. In addition, ZO-1 and ZO-2 shifted to the nucleus during hypoxia *in vitro*, a relocation that was accompanied by increased paracellular permeability [43]. Recent studies have also revealed an important role of transforming growth factor (TGF)-β-signaling in expression of TJ proteins claudin-5, occludin and ZO-1 [44]. These studies showed that peripheral inflammatory pain caused a reduction in serum TGF-β1 and protein expression of the TGF-β receptor, activin receptor-like kinase-5 (ALK5), in the brain; changes were accompanied by increased expression of TJ proteins and increased paracellular permeability of the BBB [44]. The same effects were produced by pharmacological inhibition of ALK5, which indicated that TGF-β/ALK5 signaling was involved in the regulation of TJ protein expression and/or their spatial distribution [44]. Also, oxidative stress produced during hypoxia and reoxygenation mediated an increase in BBB paracellular permeability, probably because of alterations in the localization of occludin, with movement of occludin away from the TJ [45]. Protein kinase C (PKC) is involved in control of TJ expression in BECs and it was shown that PKC isoenzyme nPKC-theta signaling mediated TJ protein rearrangement, resulting in increased BBB paracellular permeability [46]. A study on cell culture - induced changes in the blood-brain barrier transcriptome in mice by qPCR has revealed that there was a dramatic drop in the relative amount of mRNA for claudin 5 and occludin in single cultured cells, in cells co-cultured with astrocytes and in immortalized cell line, when compared to non-cultured and freshly isolated mouse BECs [47]. This finding could explain fairly low TEER values in BEC cell cultures, when compared to TEER of BECs *in vivo*.

### Adherens Junctions

Adherens junctions (AJs) are specialized cell-cell junctions that are formed by cadherins and associated proteins into which actin filaments are inserted. Optimal function of cadherins requires association of their C terminus with catenins; cadherins bind directly to β-catenin and to p120 catenin, which can bind to α-catenin, a protein that in turn binds actin [48]. In endothelial cells, vascular endothelial (VE) cadherin is present [35,49]; however, a study has shown that barrier-forming endothelium (i.e. BECs) and barrier-forming epithelium (i.e. CPE) mainly expressed cadherin-10, while the expression of VE cadherin was scarce [50]. On the other hand, brain microvessels that do not have BBB properties (i.e. in the circumventricular organs and CP capillaries) expressed only VE-cadherin and did not express cadherin-10 [50]. Also, in the microvessels of glioblastoma multiforme tumors, which lose BBB properties, VE-cadherin was expressed instead of cadherin-10 [50]. These findings suggest that cadherin-10 has an important role in the development and maintenance of the BBB and the BCSFB. Cadherins regulate endothelial functions by direct activation of phosphoinositide 3-kinase, a signaling system that has a role in organization of the cytoskeleton and forms complexes with the vascular endothelial growth factor (VEGF) receptor 2. Thus, cadherin-mediated signaling is important for endothelial cell layer integrity and for the spatial organization of new vessels [51]. At least four catenins, β, α, χ and p120 are expressed at the BBB, with β-catenin linking the cadherin to α-catenin which binds the complex to the actin network of the cell skeleton [49]. However, a study has challenged this view, since it was unable to confirm actin binding to a preformed E-cadherin-β-catenin-α-catenin complex [52]. As mentioned above, CPE expresses cadherin-10 while CP capillaries express VE-cadherin [50]. Only two catenins, α and β, have been detected in the CP epithelium so far, with α-catenin binding to the actin network [52].

In summary, BECs and CP epithelium show many similarities in the organization of Ts and AJs; the main difference is that the CPE provides a barrier that offers lower TEER values and is less restrictive than the BBB. The molecular organization underlying that difference is probably related to expression of different claudins, since those proteins play an important role in barrier size-selectivity and selectivity to paracellular movement of ions.

## Molecular biology of transport processes between blood and brain extracellular fluids

TJs restrict paracellular diffusion across cellular layers. Thus, hydrophilic molecules cannot readily enter brain ISF or CSF by simple diffusion and must be transferred across the layer by transcellular routes. On the other hand, lipid soluble non-polar molecules can easily diffuse into lipid bilayers and thus affect the composition of cellular membranes. The later process could have a detrimental impact on brain function. Thus, the BBB and the BCSFB have, in general, a similar functional organization with regard to transport of molecules: they express various proteins in their membranes that either use carrier-mediated transcellular transport of solutes, maintaining optimal composition of the brain ISF, or use ATP-driven efflux of lipophilic molecules, the latter process having an important role in maintenance of lipid bilayers in brain cells [53].

Proteins that mediate transport of solutes not directly coupled to ATP hydrolysis belong to a superfamily of solute carriers (SLC); this family includes facilitated transporters, ion-coupled transporters and exchangers that do not require ATP. They facilitate membrane transport of monosaccharides [54], amino-acids [55], monocarboxylic acids [56], vitamins [57], nucleosides [58,59], purine [60] and pyrimidine [61] bases, ions and amphipathic molecules (organic anions and organic cations). The second superfamily consists of ATP-binding cassette (ABC) proteins that directly couple efflux transport of molecules from a lipid bilayer against the concentration gradient to ATP hydrolysis [53]. Due to the presence of ABC-transporters, a large number of solutes and xenobiotics have a much lower transfer rate into the CNS than might be expected from their lipophilicity, which is expressed as logD octanol/buffer partition coefficient at pH 7.4.

There are large dissimilarities between the BBB and the BCSF in regard to expression of SLCs and ABC transporters. Also, some of these transport proteins are expressed in both membranes of the two barriers, in the one that faces brain fluids and in the one that faces blood/CP ISF; other transport proteins are inserted into either the luminal or abluminal membrane only.

### Glucose transporters

Glucose is the principal energy source for mammalian brain and a continuous supply of this substrate is essential to maintain normal cerebral function [62]. The brain rapidly catabolizes glucose, which creates a downhill gradient for this hexose from blood towards the brain ISF and glucose transport into brain is mediated by facilitative glucose transporter proteins. Delivery of glucose from the blood to the brain requires transport across the endothelial cells of the blood-brain barrier and across the plasma membranes of neurons and glia. There are also several lines of evidences indicating metabolic coupling between astrocytes and neurons, whereby glucose is used and lactate is released into the ISF by the astrocytes [63,64]. Lactate is then taken up by neurons, where it serves as an important fuel. Astrocytes appear to form the first cellular barrier that glucose faces when entering the brain and they are ideally located to provide coupling between neuronal activity and glucose uptake.

**Figure 3:**
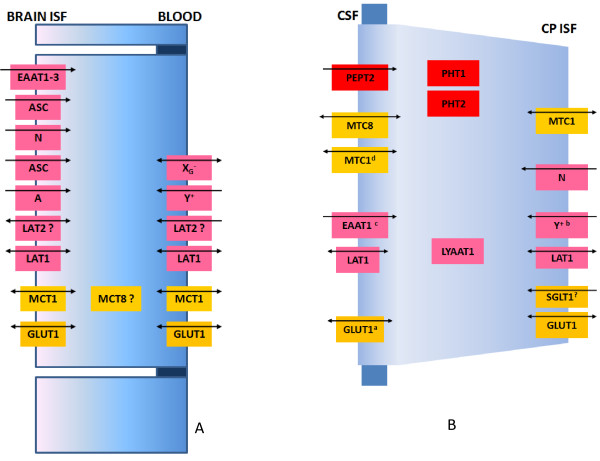
**Solute carrier transporters (SLCs) in the BECs (A) and in the CP epithelial cells (B)**. Only SLC involved in transport of monosaccharides, amino-acids, monocarboxylic acids and peptides are shown. A. A proposed model of SLCs distribution in BECs. A question mark with MCT8 transporter indicates that in BECs this transporter is detected at the transcript level, but its cellular localization is not clear. Also, there are conflicting data on LAT2 expression, also indicated by a question mark. Members of the peptide transporters family (PTR) are not present in BECs. B. A proposed model of SLCs distribution in CPE cells. A question mark indicates that there is conflicting data about presence of SGLT1 in CPE cells. Symbols in superscript indicate: ^a ^-GLUT1 is present in the apical membrane of the CPE cells, but it is much less abundant in that membrane than in the basolateral membrane; ^b ^- System y^+ ^was detected at the transcript level in CPE cells and functional uptake studies indicated that it was located in the basolateral membrane; ^c ^- Uptake studies in the rat *in vivo *indicated that EAAT1 substrates aspartate and glutamate were taken by CPE from CSF side by a saturable and stereospecific mechanism that did not show cross-inhibition with neutral amino acid. However, EAAT1 is not expressed in normal CPE in humans and is expressed in dedifferentiated CPE cells in CP tumors; ^d^-CPE cells express MCT 1, but at much lower level than BECs and cellular localization of this isoform includes both basolateral and apical membranes. CPE cells also express a lysosomal AA transporter LYAAT1, which is located intracellularly.

Several isoforms of equilibrative glucose transporters, GLUT, have been identified in the brain, which included GLUT1 (Human Genome Organization, HUGO, name SLC2A1) [65], 3 (SLC2A3) [66] and 8 (SLC2A8) [67]. GLUT 1 is a ubiquitous glucose transporter in mammalian cells and it is abundant in the brain; also it is exclusively expressed at the BBB, especially at its abluminal membrane and in CPE cells (Figure 3A, B) [62]. Thus, not surprisingly, a rat blood-brain barrier transcriptome study revealed that GLUT1 tag was within 15 of the most abundant tags enriched in rat brain microvessels, together with tags that corresponded to mRNA encoding P-glycoprotein (P-gp), transferrin receptor and the thyroid hormone transporter Oatp1c1. It was also the most abundant tag when compared to tags identifying other solute-carrier family members, indicating the importance of glucose transport at the BBB for brain homeostasis [39]. With regard to this study, it should be noted that at least several tags out of those top-15 were in fact associated with genes expressed in reticulocytes (like hemoglobin β chain), which was probably due to contamination of brain microvessels with red blood cells [39]. GLUT1 has molecular weight (MW) which can range between 45 kDa (smaller MW species) to 55 KDa (larger MW species) [68].

Most parameters for GLUT1 kinetics have been determined in red blood cells and *Xenopus *oocytes at subphysiological temperatures (20°C) using zero-trans flux estimation that revealed Km for glucose 1.6-4.6 mM and 16.9-26.2 mM (equal exchange method) [69]. However, a recent study using a multicompartmental data analysis on BECs in culture revealed a Km of 1.5-3.5 mM [70]. The larger MW species are present in microvessels [71], the smaller species are present in neurons and glial cells and the intermediate species in the CPE [72]. The different molecular weights are associated with differences in N-linked glycosylation and could also affect affinity for glucose. The other functional effects of different glycosylation states are not clear although there is evidence suggesting that they are involved in GLUT1 trafficking and substrate affinity.

GLUT 1 expression is controlled by the hypoxia-inducible factor 1 (HIF-1), which is a key regulator in cellular adaptations to a decrease in partial pressure of oxygen [73]. HIF-1α protein is unstable under normoxic conditions and is constantly degraded by activity of prolyl hydroxylases [74]. During hypoxia, HIF-1α protein is stabilized by a decrease in activity of prolyl hydroxylases, binds to its binding partner HIF-1β, and translocates to the nucleus to bind to hypoxia-responsive cis-elements [75]. Through this mechanism, HIF-1 activates multiple genes involved in angiogenesis and metabolism, including those genes that regulate glucose uptake and utilization [73]. It has been shown that transient brain ischemia causes an increase in HIF-1 "downstream" genes, including GLUT1 expression [76]. However, it is not clear if this signaling pathway plays any role in controlling GLUT1 expression under normoxic conditions. A recent study revealed that in BECs in primary culture, sodium-glucose cotransporter (SGLT) - mediated glucose uptake was induced during ischemia-like conditions *in vitro *and was also induced during permanent middle cerebral artery occlusion *in vivo *[77].

Importance of GLUT1 for the brain homeostasis can be observed in a rare genetic disease known as GLUT1 deficiency syndrome [78] that is caused by heterozygous mutations of the GLUT1 gene and is inherited as an autosomal recessive trait [79]. In this condition brain development and function are severely affected, with microcephaly and number of other CNS symptoms/signs, including developmental delay, intellectual disability, spasticity, ataxia, dysarthria and myoclonus [80,81]. Similar findings were observed after heterozygous mutations of the GLUT1 gene in mice (GLUT1^+/- ^mice), a condition that was associated with reduced expression of GLUT1 in BECs. Those animals showed signs similar to GLUT1-deficient patients, including microcephaly, seizures, incoordination, and spasticity [82].

It has been also postulated that GLUT 1 downregulation may be linked to neuronal deficits in Alzheimer disease (AD). Mooradian *et al*. in 1993 showed that cerebral cortices from AD patients contained less GLUT1 protein than controls [83]. It remains unclear whether reduced expression of GLUT1 in cortical samples of AD patients was caused by reduced demands of affected tissue or whether reduced glucose availability could be one of the causes for neuronal degeneration. Regional glucose uptake studies have demonstrated that individuals diagnosed with aging-associated cognitive decline had reduced glucose uptake in several cortical regions [84]. Mosconi *et al*. [85] suggested that reductions of glucose uptake by the hippocampus can predict cognitive decline associated to AD before clinical diagnosis. However, given the kinetic properties of GLUT1 [70], even if GLUT1 expression is reduced at the BBB, it should be associated with sufficient downhill transport of glucose to provide enough fuel to support neuronal activity.

It appears that GLUT1 expression at the BBB is also influenced by astrocytes and other glial cells, so that when the brain needs more glucose, GLUT1 expression in the BECs becomes upregulated. Regina *et al*. [86] found that treatment with conditioned medium obtained from glucose-deprived astrocytes increased endothelial GLUT1 expression and glucose uptake in rat BECs; however, no change in GLUT1 expression was observed in endothelial cells treated with astrocyte-conditioned medium when astrocytes were maintained under normoxic conditions. This indicates that hypoxic astrocytes release humoral factors that upregulate GLUT1 expression in BECs. Further insight into a proposed mechanism was provided by Yeh *et al*. [87] who found that conditioned medium from rat C6 glioma cells under hypoxia up-regulated glucose GLUT1 expression in rat BECs, whereas conditioned medium from C6 cells under normoxia caused no significant effect. This effect is likely to be mediated by VEGF, which is also a HIF-1 "downstream" gene [73]; when C6 cells were transfected with VEGF small interfering RNA that diminishes VEGF mRNA expression, it was found that conditioned medium from transfected cells under hypoxia no longer up-regulated GLUT1 expression in BECs and that a similar effect was observed when VEGF-neutralizing antibody was added to the hypoxic conditioned medium [87]. Interestingly, rat BECs in primary culture often express GLUT3, a transporter that is present in neurons but not in brain capillaries *in vivo *[86], which may be a sign of dedifferentiation of BECs in culture.

Glucose in the CSF is about 50-60% of plasma glucose, which creates a downhill gradient towards the CSF. It has been shown that there was a net glucose transfer from the fluid in CP capillaries to the CSF during *in situ *perfusion of sheep CP and that this process was Na^+^-independent [88].

Immunocytochemical studies revealed diffuse GLUT1 immunoreactivity in rats, mice and rabbits [89,90] with the basolateral CPE membrane being stained more intensively (Figure 3B). Other studies could not confirm that GLUT3 [91] and GLUT2 (SLC2A2) [92] were present in the CP epithelium. An immunogold electron microscopy study has revealed very dense staining for GLUT1 in CP capillaries, while staining of CPE was less intense [93]. Interestingly, this study found that while the basolateral membrane showed staining for GLUT1, it was almost absent on the apical membrane [93], indicating low expression. The high affinity hexokinase 1 is abundant in the CPE [91], which may indicate that glucose taken up by GLUT1 is used largely to satisfy the high metabolic demands of the CPE. Reports of expression of the sodium-dependent glucose transporter 1 (SGLT1) are conflicting in the CPE (Figure 3B).

In conclusion, there is a difference between the functional roles of glucose transporters in the BECs and in the CPE; the former provides transcellular flux of glucose towards brain ISF, which is vital for providing the brain with its main fuel; the latter appears to be more important for supplying glucose to support the CPE metabolic demands.

### Amino acid transporters

Brain requires several essential amino acids (AA) for protein synthesis; although the rate-limiting step in brain uptake of circulating amino acids is BBB transport [94], under normal physiologic conditions the synthesis of brain proteins is not rate-limited by the availability of amino acids [95]. It was revealed that the influx of amino acids from blood-to-brain approximates the rates of amino acid incorporation into brain proteins [94].

Most essential AAs are neutral, with long or bulky chains and are substrates for some of the system-L amino acid transporters (LAT) [96]. It is believed that LAT1 (SLC7A5) is the main AA transporter at the BBB; immunohistochemical analyses have shown that the LAT1 was expressed in the BECs in rats in the luminal and abluminal membranes (Figure 3A). It has been shown that, in fact, LAT1 activity is induced in Xenopus oocytes by cloned cDNA from mouse encoding 4F2 light chain (4F2lc), but its trafficking and insertion into the cell membrane depended largely on co-expression with 4F2 heavy chain (4F2hc) as 4F2lc-4F2hc covalent complex (which was also known as CD98 membrane antigen) [97]. This underlines the importance of 4F2 heavy chain in bringing and inserting LAT1/4F2lc into the plasma membrane. Human and rat 4F2hc when inserted into membranes alone induce so-called y+ L-like activity (sodium-independent transport for basic amino acids, and sodium-dependent transport for neutral amino acids). In contrast, transient transfection of rat 4F2hc in Chinese hamster ovary cells results in an increase in L-isoleucine transport with characteristics of system L [98,99]. Thus, it appears that 4F2hc is essential for proper function of LAT1, but this protein itself mediates amino-acid transport. In mouse BECs 4F2hc mRNA was the most abundant among all AA transporters mRNAs, as revealed by qPCR [98].

However, RT-PCR data and kinetic analysis of [^3^H]-leucine uptake, revealed that LAT2 (SLC7A6), which has a lower affinity for this substrate, is also expressed in rat BECs in culture [100]. Kinetic analysis of amino acid transport by the brain provided data that could indicate that both LAT1 and LAT2 show affinity for small neutral AAs, alanine, serine and cysteine [101]. However, it should be noted that mouse BECs in primary cultures had significantly downregulated all mRNAs encoding AA transporters, as revealed by qPCR [47]. Some essential amino acid are cationic; these are transported from blood into brain by a Na^+^-dependent saturable carrier, system y^+ ^(SLC7A1) that is present at the luminal side of the BBB (Figure 3A) and expression of y^+ ^in BECs exceeds 38-fold expression in the whole brain homogenate [102].

Beside LAT1, several other AA transporters are present at the abluminal, brain ISF-facing side of the BECs (Figure 3A). System A (SLC38A2) (alanine preferring) was first characterized and previous kinetic studies showed that it actively transported small nonessential neutral amino acids [103]. At least four other Na^+^-dependent carriers exist at the abluminal membrane: system ASC (SLC1A5) alanine, serine, and cysteine preferring, [104], system Bo^+ ^(SLC7A3) for basic AAs [105], system N (SLC38A5) for nitrogen rich AA (glutamine, asparagine, and histidine) [106], and excitatory amino acid transporters (EAAT) (SLC1A1-3), that mediate transport of aspartate and glutamate [107]. Small AAs, alanine and serine are transported by two Na^+^-dependent transport systems that are located exclusively in the abluminal membrane [105]: the system A, which is probably the main route for Na^+^-dependent alanine transport with a Km of 0.6 mM and system ASC that also shows affinity for large neutral AA. The physiological importance of those two transport systems is unclear, but they may be related to AA efflux from the brain.

The sodium-dependent system EAAT deserves attention because it permits a net removal of glutamate from the brain. Glutamate concentration in blood is 50-100 μM [107,108]; in whole brain homogenate it exceeds 10 mM, while in the brain ISF it is normally kept below 2 μM [109]. Glutamate can exert neurotoxicity if it accumulates in the brain ISF, because, through its action on metabotropic NMDA receptors, it could lead to Ca^++ ^overload, causing neuronal injury or death [110]. Glutamate is released during neurotransmission but is normally rapidly taken up by neurons and neighboring astrocytes. However, during cerebral ischemia and/or hypoxia, this AA accumulates in the brain ISF, especially in regions that are rich in glutaminergic neurons. There are three EAATs present at the abluminal side of the BECs, EAAT1 (SLC1A1), EAAT2 (SLC1A2), and EAAT3 (SLC1A3) [107,110] (Figure 3A) and their action appears to be important to prevent excitotoxicity because they actively remove glutamate from the ISF into the BEC cytoplasm. At the luminal side of the BBB, glutamate is transported by facilitative glutamate transporter X_G_^- ^[110]. It has been demonstrated that scavenging glutamate in the blood with a glutamate-scavenging agent oxalocaetate increased the efflux of excess glutamate from the brain and reduced brain damage after closed head injury [111].

Available data on amino acid concentrations in the CSF is inconsistent, but it is clear that CSF to plasma ratios are lower than 1, ranging from <0.1 for neurotransmitters, like glycine and glutamic acid, to > 0.1 for small neutral AAs [112]. Early functional studies revealed that many neutral AA as well as glutamate and aspartate were taken across the luminal side of the CPE by a Na^+^-independent mechanism [113]. It was revealed later that the CPE expresses LAT1 [114], which could be responsible for the observed Na^+^-independent AA uptake, but expression of this transporter is less abundant than expression in the BECs (Figure 3B). A second AA transporter that is abundant in the BECs, the y^+ ^AA transporter, was also found to be present in the CPE at the transcript level; however, its abundance in the CP was less than in the BECs [102]. Previous functional uptake studies revealed that arginine and leucine were taken up by the blood side of the sheep CP by a separate transport process that did not show any cross-inhibition with neutral amino acids [115]. Uptake studies have indicated that CPE expresses system N, while the transport activity for small neutral AA (mediated by systems A and ASC) was absent [116] (Figure 3B), which confirms finding by Preston and Segal [115] that uptake of A and ASC substrates by isolated perfused CP was very low. Both choroid plexus epithelium and ependymal cells lining the ventricles express the lysosomal amino acid transporter(LYAAT-1) that mediates H^+ ^co-transport with a stoichiometry of 1 H^+^/1 amino acid [117] (Figure 3B); however its role is not very clear. LYAAT-1 plays a role in the active efflux of amino acids from lysosomes and in the CNS it is also abundantly present in neurons [117].

It has been shown, using ventriculo-cisternal perfusion in rat, that accumulation of aspartate and glutamate by the choroid plexus from CSF side was saturable, stereospecific, not inhibited by neutral amino acid analogues, and shared by both aspartate and glutamate [118]. A recent study in humans revealed that CPE, contrary to the BBB, does not express EAATs [119], which suggests that it does not normally play an active role in removing those excitatory neurotransmitters from brain extracellular fluid. However, de-differentiated CP cells, seen in CP tumors, express EAAT1; this feature distinguishes neoplastic from normal CP and could be used as a helpful diagnostic tool [119].

### Monocarboxylate transporters

As noted above, the CNS is an obligate glucose consumer that depends almost entirely on the supply of glucose from the systemic circulation. However, several findings suggest that glial cells and neurons do not use glucose as a fuel to the same extent: astrocytes take up glucose that is transported across the BECs and use it for the glycolysis, producing lactate that is released into the ISF and subsequently taken up by surrounding neurons [120]. Also, evidences suggest that neurons during development use lactate as an important source of energy during neuronal migration, since *in vivo *blockade of lactate transport in the brain over postnatal day 1-3 in mice induced a cytoarchitectonic disorganization in the parietal cortex that was likely due to a disturbance of cortical neuronal migration and an increased neuronal cell death [121]. Lactic acid has a pKa of 3.9, thus it exists almost entirely as the lactate anion at physiological pH. Both the proton and the lactate or other monocarboxylate anions require a specific transport mechanism to cross cell membranes, which is provided by proton-linked monocarboxylate transporters (MCTs) [122]. Fourteen MCTs have been identified so far [123]. Four MCTs are present in the brain: MCT1, MCT2, MCT4 and MTC8, which are selectively present in distinct cell types and membrane domains [124]. MCT4 is expressed in astrocytes and its main role is to export lactate produced during glycolysis into the ISF; from there lactate is transported into neurons by MCT2 [124]. BECs express MCT1 (SLC16A1) at both luminal and abluminal membranes and also in intracellular organelles (Figure 3A) [125] and this transporter has a fairly high affinity for lactate when compared to other MCTs (Km 3.5 mM, [124]). MCT8 (SLC16A2) mRNA and protein are also expressed in cerebral microvessels [126]. Human MTC8 transporter mediates transport of thyroid hormones and the importance of transport for thyroid hormone signaling was revealed by the discovery that inactivating mutations in the human monocarboxylate transporter-8 (MCT8) cause Allan-Herndon-Dudley syndrome, an X-linked developmental disorder characterized by hypotonia, spasticity, muscle weakness, neurological problems, and cognitive impairment due to thyroid hormone deficiency in the CNS [127]. In humans, plasma lactate is below 1 mM under normal physiologic conditions while in the brain ISF it is above 3 mM [64]. Under those conditions the MCT1 at the BBB probably pays a role in lactate removal from the brain ISF to the blood, to avoid its accumulation in the brain. However, during starvation, when following a ketogenic diet or under hypoxic conditions, plasma lactate and ketone bodies increase so the gradient across the BBB could change. It has been shown that diet-induced ketosis in rats caused a substantial upregulation of MCT1 at the BBB, associated with an increased extraction of plasma ketone bodies by the brain [128]. Interestingly, the rat BBB transcriptome study has revealed that a tag that identified MCT7 (Slc family 16 member 6) was the second most abundant tag in the microvessel SAGE catalog, with abundance that was only slightly below that of GLUT1 [39].

CPE primarily expresses MTC8, which is located on the apical surface and it is believed to be involved in thyroid hormone transport [126] (Figure 3B). It also expresses MTC1, but at much lower level than BECs and cellular localization of this isoform includes both basolateral and apical membranes [129], while MTC2 transcripts were not found in the CPE [130].

### Peptide transporters and receptors

The delivery of peptides to the brain has important physiological and clinical implications, because in many neurodegenerative diseases it has been found that the application of various growth factors/neuroactive peptides may protect neurons and/or stimulate neuronal growth and repair and, thus, improve outcome for neurological disease. Peptide-based amyloid-β (Aβ)-aggregation inhibitors have been shown to decrease the deposition of Aβ in transgenic mouse models of Alzheimer's disease [131]. Also, nerve growth factor (NGF) showed the ability to reduce neuronal degeneration in animal models of Alzheimer's disease [132]. *In vitro *and *in vivo *data suggest that treatment with neurotrophic factors such as NGF, glial cell line-derived neurotrophic factor (GDNF), brain-derived neurotrophic factor (BDNF) and several neurotrophins (NTs) could induce survival of specific neuronal populations in Huntington's disease [133]. Treatment strategies aiming to regenerate existing dopaminergic neurons in Parkinson's disease by applying GDNF, BDNF, IGF and NT-4/5 have also been attempted [134,135].

However, blood-to-brain transfer of intact peptides remains controversial. Peptides cannot use AA transport systems for facilitative transport because of the existence of the peptide bond (for a review see [136]). Even dipeptides that contain LNAAs do not show measurable affinity for facilitative transport by LAT1 at the BBB [137]. However, there are specific transport systems that mediate transport of peptides. The peptide transporters that belong to the peptide transporter (PTR) family are solute carrier proteins (SLC15A) responsible for the membrane transport of di- and tripeptides [138]. Another peptide transporter family (PTS), that contain at least 9 members (PTS1-9) mediate transport of larger peptides (more than 3 AAs in chain) and in many tissues act primarily as an efflux pump, removing lipophilic peptides from cellular membranes. PTR family consists of four members, two peptide transporters PEPT1 and 2 (SLC15A1-2) and two histidine transporters that also transport dipeptides (PHT1 and 2, SLC15A3-4). PTRs couple substrate movement across the membranes to movement of protons down an inwardly-directed electrochemical proton gradient [138].

Early studies have shown that arginine vasopressin (AVP) [139], enkephalins [140,141], delta-sleep-inducing peptide (DSIP) [142] and luteinizing-hormone-releasing hormone (LHRH) had a measurable volume of distribution in the guinea pig brain after *in situ *perfusion but the rates of blood-brain transfer were 10^3^-10^4 ^fold lower than rates of carrier-mediated amino-acid transport. Tetrapeptide tyrosine melanocyte-stimulating inhibitory factor 1 (Tyr-MIF-1) was the first peptide shown to pass from blood to the brain by a saturable system [143]. Although there is no evidence so far that any of the PTR four members, that could mediate efflux transport of di- and tri-peptides are present in the BECs, these cells probably express at least some PTS members located at the abluminal side that mediate efflux transport of several small peptides from the brain ISF: enkephalins, Tyr-MIF-1, arginine vasopressin (AVP) and LHRH [144]. For example, pituitary adenylate cyclase-activating polypeptide (PACAP), which has neuroprotective effects against ischemia, can pass across the BBB, but its efflux, which is mediated by PTS-6, severely restricts its net entry into the brain ISF. However, when PTS-6 expression in BECs was inhibited by antisense targeting, brain accumulation of PACAP increased significantly [145], which indicates that the main role of this transporter is efflux transport. Thus, it appears that BBB transport system for peptides could be involved in impeding blood-to-brain ISF transfer of intact peptides. The brain delivery of peptides is further impeded by the existence of various enzymes in BECs that modify AA side chains or hydrolyze peptide bonds. These enzymes include γ-glutamyl transpeptidase, aromatic acid decarboxylase, dipeptidyl(amino)peptidase IV, and aminopeptidases A and N [146]. However, it has been shown that some neuropeptides, when present in capillaries, could be transferred to the brain ISF in intact form, like DSIP [147].

Larger peptides and proteins that have receptors present at the luminal side of BECs could use receptor-mediated transcytosis to pass across the BBB and that mechanism was revealed for insulin [148], transferrin [149], certain cytokines [150], leptin [151,152], immunoglobulin G [153], and insulin-like growth factor [154]. It seems that Aβ could also pass the BBB by receptor-mediated transcytosis. This peptide (MW ~4500 Da) is bound in plasma to several proteins, including albumin, apolipoprotein E (apoE), apolipoprotein J (apoJ), transthyretin (TTR), α2-macroglobulin (α2M) and low-density lipoprotein receptor related protein-1 (LRP1) [155-157]. There is evidence which suggests that influx of Aβ into the brain across the BBB involves binding of this peptide to the receptor for advanced glycation end products (RAGE) and subsequent receptor-mediated transcytosis [158]. Advanced glycation end products (AGE) accumulate in the basement membrane of the BBB and this triggers increased expression of RAGE in the BECs, which could lead to increased blood-to-brain transcytosis of Aβ [159]. In the brain ISF, Aβ is degraded while some remains bound to apoJ, apoE and α2M [160]. Some reports suggest that expression of LRP1 has an important role in the prevention of Aβ accumulation in the brain by several mechanisms. One mechanism includes binding of brain Aβ to LRP1 at the abluminal (brain ISF-facing) membrane of the BECs *in vitro*, which leads to a subsequent transcytosis of Aβ-LRP1 complex [161]. Some of the Aβ-apoJ complexes from the brain ISF bind to LRP2 (also known as megalin), which also triggers transcytosis of this complex and subsequent brain-to-blood efflux of Aβ [162]. Aβ also binds to LRP1 in plasma; taking into account the abundance of LRP1 in plasma when compared to amount of circulating Aβ, this binding provides a peripheral 'sink' for plasma Aβ [160]. However, a key point in this hypothesis is challenged by *in vivo *findings of Ito and colleagues [163], who revealed, using the brain efflux index technique in mouse that simultaneous injection of receptor-associated protein (RAP) with radiolabelled Aβ failed to cause inhibition of Aβ efflux transport across the BBB. Since RAP is a chaperon protein which inhibits the ligand interactions with LRP1, the authors conclude that LRP1 interaction with Aβ is not essential for brain efflux of Aβ and that this efflux is LRP1 independent [163]. This finding was challenged [160], by a group who claim that Ito and co-workers have not performed control experiments to determine ^125^I-Aβ integrity prior to its use and/or at the end of the experiment in brain extracts.

In adsorptive endocytosis, the interaction of a glycoprotein, or positively-charged peptide, with glycoproteins or negatively-charged regions of the BECs causes adsorption of the molecule to the surface of the BECs. This in turn causes internalization of the molecule into the BEC [164]. However, it is not clear what determines the fate of those vesicles; they could be delivered to the Golgi, or lysosomes, or they could move across the cytoplasm and fuse with the abluminal membrane [165]. An alternative strategy to enhance peptide delivery to the brain is modification of amino acid side chains in small peptides (150-500 Da) to improve liposolubility (ideally a log octanol/water partition coefficient should be 0.5-6.0), to enable these peptides to diffuse across the BEC membrane [166]. Although it has been proposed that peptides with MW > 400 Da cannot cross the BBB [167], so far there is no clear evidence that an absolute MW cutoff point exists for crossing the BBB. However, this strategy faces another obstacle, because with increasing lipid solubility sequestration by liver and binding to plasma proteins also increases, which decreases the half-life of a peptide in plasma and reduces availability for interaction with BECs. Also, free diffusion is often followed by proteolytic cleavage in the BECs or active efflux from the BECs by P-gp or PTSs [144]. For example, blood-to-brain transport of Tyr-MIF-1 and the enkephalins is very limited because of the action of PTS1 in the abluminal membrane of BECs, which mediates efflux of those peptides [168]. Also, P-gp mediates efflux transport of several opiate peptides and inhibition of efflux pumps was accompanied by a several-fold increase in peptide accumulation by the brain [169].

Another alternative strategy is to use receptor-mediated transcytosis through the BBB for drug delivery to the brain. This strategy, known as a "Trojan horse", includes conjugation of different peptides that have very limited delivery to the brain to monoclonal antibodies against one of the BBB peptide receptors, like the transferrin receptor [170]; binding of the antibody to receptor triggers endocytosis, as explained above. However, these receptors are not brain specific and are widely expressed in peripheral organs, a fact that limits their applicability for brain-targeting. A recent study used phage display in an *in situ *brain perfusion model to screen for peptide ligands that bind specifically to brain endothelium [171]. Using this strategy, new peptide ligands were identified that showed significant binding to human brain endothelium but not to other human endothelial cells, so they may be used for specific targeting of drugs to the blood-brain barrier [171].

BECs are not only involved in transport/transcytosis of peptides/proteins, but also as a target for various bioactive peptides and in synthesis of neuroactive peptides. It has been shown recently in a study on AVP-deficient Brattleboro rats, that BECs produce and secrete several chemokines after brain injury; this production is under the synergistic control of AVP and TNF-α [172]. The AVP effects were mediated by c-Jun N-terminal kinase (JNK), a kinase that has increased activity in BECs in response to injury [172].

Contrary to the situation at the BBB, several lines of evidence indicate that members of the PTR family of proton-coupled peptide transporters are expressed at the BCSFB; this includes PEPT2, which is expressed in the CP membranes [173] and PHT1 [174] (Figure 3B). PEPT2 is a proton-coupled oligopeptide transporter and it is abundant in epithelial layers, including kidney, where it plays an important role in renal reabsorption of di- and tri-peptides [138,175]. All evidence available so far suggest that PEPT2 is located in the apical, CSF -facing side, and it was responsible for 95% of dipeptide (glycylsarcosine - GlySar) uptake by isolated CP that was incubated in artificial CSF containing GlySar [173]. Wild-type mice had greater choroid plexus concentrations of GlySar and a 5-fold greater CP/CSF ratio when compared to PEPT2-null mice [175] and it was located at the apical side of the rat CPE in primary culture [176]. PEPT1 and PEPT2 have wide affinity for di-and tripeptides with more than 8000 different substrates identified so far [138]. Apart from CPE, in the CNS PEPT2 is located in ependymal cells lining cerebral ventricles [177]. Thus, the likely function of this transporter is to clear di- and tri-peptides from the CSF. An intense hybridization signal for PHT1 was found in the brain, especially in the hippocampus and cerebellum, while the signal in the CP was weaker [174]. PEPTs also transport a number of peptidomimetics, so the presence of this transporter at the apical membrane of the CPE could severely restrict entry of blood-borne peptidomimetics into the CSF. The CPE is also involved in receptor-mediated endocytosis of peptides. It has been shown that transport of blood-born leptin to the CSF involves leptin binding to LRP2 (megalin) in the CPE and transcytosis of the LRP2/leptin complex through epithelial cells [178]. The CPE also plays a role in clearance of Aβ from the CSF and it appears that LRP2 is involved in this process, since it has been shown that AD patients have reduced levels of LRP2 at the CP, which may decrease efflux of Aβ from the CSF and could be, therefore, one of the causes of increased brain levels of Aβ [179]. Another receptor belonging to the same LDL receptor gene family appears also to be involved in Aβ clearance from the CSF: LRP1 is normally confined to the CPE cytoplasm where it binds Aβ; however, exposure to lead causes protein kinase C-mediated relocation of LRP1 and disrupts normal clearance of Aβ from the CSF, leading to its accumulation in the CPE [180].

The CPs are important not only in clearance of peptides from the CSF and delivery of blood-borne peptides to the CSF, but also as a target for a number of hormones. Atrial natriuretic peptide (ANP) binds to its receptors in the CPE, which generates cGMP; this second messenger alters ion transport, thereby slowing CSF production [181]. The presence of natriuretic peptide receptor (NPR) A and NPR B, both containing a guanylyl-cyclase intracellular domain, was confirmed by immunostaining in CPE, and also in some ependymal cells in adult Sprague-Dawley rats [182]. Rats with congenital hydrocephalus had a lower number of binding sites for radiolabelled ANP in the choroid plexus, as compared to the control rats, indicating that this could be one of the pathophysiological mechanisms underlying excessive CSF production [183]. The CPE is also one of the extra-hypothalamic sources of AVP [184]. The regulation of choroidal AVP synthesis is similar to that observed in the hypothalamus and it has been shown that chronic hypernatremia increases the expression of AVP in the CP [185]. It also appears that a well-known inhibitory effect of centrally-released angiotensin II on CSF production by the CPs is mediated by AVP production in the CP and its paracrine action on V1 receptors present in the CPE [186], which involves V1 receptor-mediated decrease in Cl- efflux from epithelial cells and consequent reduction in CSF formation [187]. An important role of CP-born bioactive peptides is proposed in brain recovery after traumatic brain injury (TBI). It is believed that upregulated growth factors and neurotrophins produced by the CPs and by the ependymal layer are brought by the CSF bulk flow to brain regions close to the ependymal layer and those factors could be important for neural restoration through enhanced neurogenesis and angiogenesis after TBI [188].

An important feature of CPE, that is not present in the BECs, is a synthesis of transthyretin (TTR), which functions as a carrier for thyroxin and retinol-binding protein [189]. It also sequesters Aβ peptide, and TTR levels in the CSF appear to be inversely correlated with Alzheimer's disease (AD) onset and progression. TTR, thyroxin-binding globulin (TBG) and albumin form a "buffering" system for plasma L-thyroxin because of their overlapping affinities for that hormone. CPs have the highest concentration of transthyretin (TTR) mRNA in the body and the percentage of TTR to total protein synthesis in choroid plexus exceeds 10% [189]. However, absence of TTR in genetically-modified mice did not affect delivery of T4 to the brain [190]. 5-α-dihydrotestosterone treatment increased TTR protein levels in CPE cells in primary culture and induced TTR transcription in these cells *via *an androgen receptor-independent pathway [191]. On the other hand, treatment with 17β-estradiol increased expression of TTR in CPs *in vivo *at both transcript and protein levels via an oestrogen receptor α-dependent pathway [192,193]. Also, expression of TTR both *in vitro *and *in vivo *was up-regulated by treatment with progesterone, which involves a progesterone receptor-mediated mechanism [194]. These findings could at least partially explain mechanisms involved in protective effects of progesterone and estradiol against the onset of AD.

### ABC-transporters, organic anion/cation transporters and organic anion transporting polypeptide expression at the BBB and the BCSFB

The family of ATP-binding cassette (ABC) transporters is divided into subfamilies: the multidrug-resistance proteins or P-glycoproteins (Abcb subfamily, HUGO names ABCB1-11), the multidrug resistance-related proteins MRPs (Abcc subfamily, HUGO names ABCC1-5) and the breast cancer-resistance protein (BCRP, HUGO names ABCG1-8) [195]. Their substrates range from small ions to large polypeptides and transport occurs against steep concentration gradients using energy that is provided by ATP-hydrolysis [196]. Transport of amphipathic molecules (i.e. organic anions) is sodium-independent and mediate by transport proteins that belong to two SCL families, the organic anion/cation transporter family (OATs - SLC22) and the organic anion transporting polypeptides family (OATPs - SLC21). Members of the SLC21 family mediate transport of large, amphipathic solutes such as bile salts, thyroid hormones, leukotriene, and various steroids conjugates and xenobiotics [197]. OATs accept smaller and more hydrophilic substrates than those carried by members of the SLC21 family, including neurotransmitter metabolites, cAMP, cGMP, and xenobiotics such as para-aminohippuric acid, β-lactam and sulfonamide antibiotics, non-steroidal anti-inflammatory drugs, antiviral drugs, antidiuretics, antiepileptics, methotrexate [197]. Substrates for OCTs include neurotransmitters (5-HT, dopamine), choline, tetraethylammonium ion, cimetidine, N1-methylnicotinamide [198].

**Figure 4:**
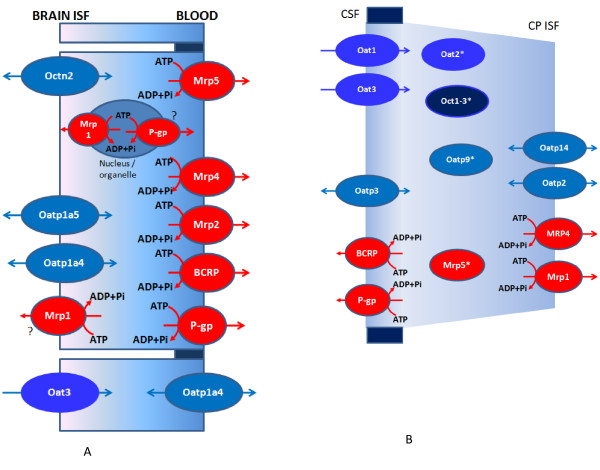
**Distribution of ABC-transporters, organic anion/cation transporters and organic anion transporting polypeptide expression in the BECs (A) and in the CP epithelium (B)**. Members of the ABC family are presented in red, while SLC members are presented in various tones of blue. Some data indicate that in the BECs Mrp1 and P-gp may also be present in organelles and nuclear envelope. Membrane localization of Mrp1 in BECs is not completely clear, with some reports indicating that it is present at the luminal side, while others indicating that it is scarce and probably located at the abluminal side. Mrp5 was detected in the CPE cells at the transcript level, but there are no functional or immunocytochemical data so far, indicating its cellular localization (asterisk). The same stands for Oat2, Oct1-3 and Oatp9.

Given the mechanism of action of particular ABC transporters, the precise localization of these proteins at the BBB and BCSFB is essential for understanding their role in physiology and in drug delivery to the brain. For example, a luminally-located P-glycoprotein, which is quantitatively the most important ABC transporter at the BBB, would mediate efflux transport of its substrates from the luminal membrane back to blood, which would impede influx of substrates to the brain. On the other hand, abluminally located P-gp would mediate transport of substrates from the abluminal membrane into the brain ISF, thereby facilitating influx of substrates to the brain. In brain capillaries, P-gp is predominantly and abundantly expressed in the luminal membrane [199] and it mediates efflux of substrates back into the blood after they initially diffuse into the endothelial cell membrane (Figure 4A). By this action, P-gp restricts penetration of its substrates into the brain. A report has suggested that endothelial P-gp is expressed at the nuclear membrane of rat brain microvessel endothelial cell line RBE4 [200]. In rodents, two multidrug resistance proteins are encoded by the genes Mdr1a and Mdr1b and only Mdr1a is found in endothelial cells [201]. Studies using P-gp knockout mice have mainly contributed to the view of P-gp as the main gatekeeper at the BBB [202]. Both SAGE analysis of the rat BBB transcriptome and qPCR analysis of mouse BBB transcriptome revealed that P-gp mRNA was highly expressed in brain microvasculature [39,47]. The expression of MRPs is less clear and there are many conflicting reports: some authors suggested that BECs express multidrug resistance-associated protein Mrp1 (for the review see [203]) at the luminal side, while others revealed by immunofluorescence staining that this protein is scarce at the BBB and localized abluminally [199] (Figure 4A). However, MRP4, MRP5 and probably MRP2 are located on the luminal membrane of BECs (for reviews see [203,204]); MRP3 has only been detected in capillaries from brain tumors [205]. Breast cancer-resistance protein (BCRP, ABCG2) is expressed at the luminal membrane of human BECs [206] (Figure 4A) and its substrate specificity partially overlaps with that of P-gp. Data suggest that after P-gp, BCRP is the second most abundant ABC transporter expressed in human BECs [207]. In rodents, Oatp1a4 (Slc21a5, old protein name Oatp2), Oatp1a5 (Slc21a7, old protein name Oatp3) and Oatp1c1 (Slc21a14, old protein name Oatp14) are expressed at blood-brain interfaces with Oatp1a5 being located primarily abluminally and Oatp1a4 on luminal and abluminal membranes [126,203,208]. In humans OATP1A2 (SLC21A3, old protein name OATP-A) and OATP2B1 (SLC21A9, old protein name OATP-B) are localized at the luminal membrane of BECs [209]. At the rodent BBB, Oat3 (Slc22a8) is predominantly localized at the abluminal membrane [210], while OAT3 (SLC22A8) and OAT1 (SLC22A6) are found in epithelial cells of the human CP [211], but their precise localization is not clear. Electrogenic organic cation transporters (OCTs) are expressed in rodent and human neurons and glial cells and not in BECs in humans [212]. The proton gradient-driven OCTN2 (SLC22A5), which mediates transport of carnitine, is expressed in the abluminal membrane in bovine BECs [213].

Many of those transporters have also been identified in the CPs at the transcript or protein level or by functional transport studies [214]. P-gp expression in the human and rodent CP has been detected [215,216], but other research groups have found P-gp in the CP to be scarce or undetectable [217,218]. Studies that detected it in the CP reported that P-gp was located at the apical (CSF-facing) side and in sub-apical cell compartments [216] (Figure 4B), which means that P-gp transports substrates back into the CSF. So, the direction of P-gp-mediated transport at the BCSFB appears to be opposite to that at the BBB. The most abundant efflux transporter in the CP is Mrp1 (ABCC1) and it is located basolaterally [216] (Figure 4B); MRP4 is also present in the basolateral membrane of the human CPE cells [218]. Presence of mRNA for Mrp5 (ABCC5) in the CP has also been revealed [219]. BCRP is located on the CSF side of CPE cells in mice [220]. Cellular localization of two Oatps in CP has been confirmed by immunochemical studies: Oatp1a4, (Slc21a5, old protein name Oatp2) is located at the basolateral membrane, while Oatp1a5 (Slc21a7, old protein name Oatp3) is located on the apical membrane [221,222], with Oatp2 being probably the most abundant Oapt in the CP [223]. Oatp2b1 (Slc21a9, old protein name Oatp9) and Oatp1c1 (Slc21a14, old protein name Oatp14) were detected in CP at the transcript and protein level; precise cellular localization of Oatp9 is unknown [224], while Oatp14 appears to be located primarily at the basolateral membrane (Figure 4B), and is involved in thyroid hormone transport [126]. Members of the Slc22a gene family, OAT1 (SLC22a6) and OAT3 (SLC22a8) are located at the apical side of the CPE [225], while expression of OAT2 mRNA in the CP was confirmed but there are no data about cellular localization [224]. The presence of Octs 1-3 has been confirmed by RT-PCR but there are no further data on their cellular localization.

Overall, the two important differences between the BBB and the BCSFB with regard to expression of these transporters are: P-gp is present at the blood-facing side in the BECs and it is the most abundant transporter at the BBB, while in the CP P-gp is expressed predominantly on the apical, CSF-facing side of the CPE cells and it appears that the P-gp function in the CP is not that critical for brain homeostasis, since the amount of that protein in the CP is negligible (~0.5%) when compared to its amount in BECs [217]. On the other hand, at the BBB Mrp1 is fairly sparse while in CPE it is probably the most abundant transporter, exceeding by at least 200-fold that in the BECs [217].

Since P-gp appears to be the main gatekeeper at the BBB, a very important observation was that xenobiotics, environmental toxins and pollutants, mediators of inflammation and even the neurotransmitter glutamate could affect expression of P-gp in the BECs, thereby reducing or increasing drug delivery to the brain [213]. Briefly, exposure of rat BECs to xenobiotics or pollutants that are ligands to androstane receptor (CAR) or to pregnane-X receptor (PXR) causes activation of these two receptors and then activated CAR/PXR translocate to the nucleus to increase P-gp gene expression [226,227]. A practical consequence of this mechanism is that treatment with drugs that are P-gp substrates could reduce delivery of other P-gp substrates to the brain. Inflammatory signals have more complex effects on P-gp expression: initially they cause a loss in P-gp activity that is followed by delayed increase in activity and expression [228]. Rapid and reversible loss of P-gp transport function is not accompanied by change in protein expression and it involves binding of ligands (endothelin - ET and tumor necrosis factor alpha - TNFα) to toll-like receptor-4 (TLR4) or TNF-α receptor 1 (TNF-R1), which is followed by activation of nitric oxide synthase (NOS) and protein kinase C (PKC). NOS and PKC modify activity of the existing P-gp. Delayed induction of P-gp expression includes signaling via TNF-α [229] and ET-1, but in this case a signaling cascade activates nuclear factor-κB (NF-kB), which is a ubiquitous transcription factor that controls the expression of genes; NF-kB then translocates to nucleus affecting gene expression [230]. TNF-α-mediated signaling includes not only an increase in expression of P-gp, but also reduces the amount of Mrp2 and Mrp4 proteins [229]. Also, it was revealed that diesel exhaust particles (DEP) that can be found in polluted air could increase expression of P-gp, Mrp1, Mrp2 and BCRP in BECs [231]. The proposed mechanism involves TNF-α signaling, which means that chronic exposure to DEPs could cause additional oxidative and inflammatory stress for the brain; this corresponds to finding that DEPs induce inflammatory responses in microglia [232].

Another challenging area for ABC transporter research is a hypothesis that their failure could be associated with Alzheimer's disease (AD) where P-gp and Bcrp could serve as efflux pumps for β-amyloid peptide [233,234]. The existing reports are conflicting: it has been revealed that brain capillaries of Alzheimer's patients have reduced expression of P-gp [235] and increased expression of BCRP [234]; however, a recent study by confocal microscopy that quantified peak fluorescence values of cross-sectional profiles of brain microvessels, revealed expression of P-gp protein to be significantly lower in hippocampal vessels of patients with AD compared to normal individuals, whereas that of MRP4 or BCRP protein was not changed [236]. However, the same study reported that analysis of the sections at protein level *via *Western blotting or at transcript level by qPCR did not reveal significantly lower expression for either P-gp or BCRP [236].

### Ion transporters in the BBB and CP

**Figure 5:**
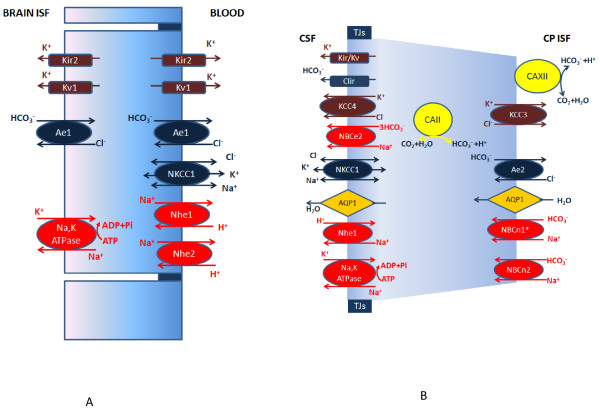
**Distribution of ion transporters and channels in the BECs (A) and in the CP epithelium (B)**. Only those transporters and channels that play a role in vectorial transport of Na^+^, Cl^-^, HCO_3_^- ^and K^+ ^are shown. These include Na^+^, K^+^-ATPase, potassium channels Kir and Kv, chloride/bicarbonate channel Clir and members of the SLC: Na^+^, HCO_3_^- ^cotransporters 1 and 2 (NBCn1 and 2), Cl^-^, HCO_3_^- ^exchangers 1 and 2 (Ae 1 (in the BBB, figure A) and Ae2 (in the CP, figure B), Na^+^, H^+ ^exchanger 1 and 2 (Nhe1 and Nhe2), K^+^-Cl^--^cotransporters 3 and 4 (KCC3 and 4, in the CP, figure B), Na^+^-K^+^-2Cl- cotransporter 1 (NKCC1, in the BBB, figure A) and electrogenic Na^+^, HCO_3_^- ^exchanger (NBCe2, in the CP, figure B). In addition, localization of two carbonic anhydrase isoenzymes in the CP epithelium (figure B), CA2 and CA12 are shown, as well as localization of aquaporin 1 (AQP1). Symbol * indicates that NBCn1 was detected in CPE, but it probably does not play a role in vectorial transport of these two ions.

There is evidence suggesting that there is a bulk flow of the brain ISF from brain capillaries towards the ventricular space and that ISF merges with the CSF; this flow takes place predominantly along perivascular spaces (for a review see [237]). This indicates that there is a constant production of a "new" ISF in the brain which contributes to total volume of the CSF. Although some of the ISF is probably generated from water produced by brain metabolism, fluid secretion by BECs appears to be an important source of this ISF [237], accounting for at least 30% of the ISF production [238]. This process is essential for maintaining correct fluid balance in the brain. Two membrane proteins that work simultaneously but at different membranes of the BECs are key regulators of net sodium and chloride transport across the BBB: Na^+^, K^+^-ATPases (ATP1 family) and the Na^+^, K^+^, 2Cl^-^cotransporter (SLC12 family). The Na^+^, K^+^-ATPase is localized on the abluminal membrane and provides a driving force for the net ion and water movement across the BBB [239] (Figure 5A); 3 alpha- (ATP1A1-3) and 2 beta- (ATP1B1-2) subunit isoforms were found in rat BECs, which means that six structurally distinct Na^+^, K^+^-ATPase isoenzymes are likely to be expressed in brain microvessels [240]. As in other tissues, the activity of this enzyme is tightly associated with cell volume regulation [241]. The Na^+^-K^+^-2Cl^-^- cotransporter is located at the luminal side of BECs [242] and its activity is regulated by PKC signalling [243]. In addition, rat brain endothelial cells express Kv1 and Kir2 potassium channels; these are probably located on both the luminal and abluminal sides of the BECs [244] (Figure 5A). Furthermore, BECs also express two ion exchangers that belong to the SLC family and are probably involved in intracellular pH regulation: chloride-bicarbonate (Cl^-^, HCO_3_^-^-) exchanger (SLC4A1) and sodium-hydrogen (Na^+^, H^+^-) exchanger (SLC4A6). Both isoforms of Na^+^, H^+^-exchanger (Nhe1 and Nhe2) are expressed on the luminal membrane, whereas chloride-bicarbonate exchanger(Ae1) is expressed at both luminal and abluminal membranes of the BECs (Figure 5A) [245]. Net flux of K^+ ^at the BBB is critical for brain homeostasis, since changes in concentration affect resting membrane potential; however, net flux of this ion across the BBB is not well understood. Both Na^+^, K^+^-ATPase and the Na^+^-K^+^-2Cl^-^cotransporter bring this ion into the BECs and at least two potassium channels exist on both sides (Figure 5A). A hypothesis suggested that there was a net K^+ ^efflux across the BBB, which contributed to low K^+ ^concentration in the brain ISF [246]. However, the CSF recovered from the brain, contains 2.5-3.0 mM K^+ ^and the CSF originates either from CP secretion or from the brain ISF, although the relative contribution of those two sources is a matter of debate. Thus, at least one of these two (CSF secreted by the CPS or brain ISF secreted by the BECs) have to be a source of K^+ ^in the CSF. CPs in fact mainly reabsorb K^+ ^from the CSF (see below). Thus, bearing in mind conservation of mass, a speculation could also be made that K^+ ^found in the CSF is, at least partially, K^+ ^that was secreted at the BBB.

Ion transport at the BBB also plays an important role in regulation of endothelial cell pH, especially bicarbonate transport driven by Cl^-^, HCO_3_^- ^-exchanger and H^+ ^transport driven by Na^+^, H^+ ^-exchanger. It has been shown that following *in vitro *loading of BECs with small acid load, HCO_3_^- ^influx was mainly responsible for the acid extrusion and it was mediated partially by Cl^- ^dependent HCO_3_^- ^transporters. However, after large acid loads, BECs removed acid almost solely by Na^+^, H^+ ^exchange, with the rate of its activity depending linearly on intracellular pH [247]. Following an alkaline load to BECs, the intracellular pH was restored by acid loading which occurred *via *Cl^-^/HCO_3_^- ^exchange [247].

Ion transporters in the CPs have been studied intensively because ion transport across the CPE drives CSF secretion, which could be considered as the most apparent and the most important function of CPs. CSF has a number of important roles in brain homeostasis, including reduction of the effective weight of the brain by being submerged in CSF, removal of waste products of metabolism, removing excess neurotransmitters and debris from the surface lining epithelium and delivering signalling molecules (for a review of CSF functions see [248]). Bicarbonate transport by the CPE directly affects the pH of CSF, which in turn affects neuronal activity in the respiratory centre in the *medulla oblongata*.

Overall, two main processes are driven by ion transporters in the CP. First, the transepithelial basolateral-to-CSF movement of sodium, bicarbonate and chloride creates a small osmotic force driving net movement of water in the same direction. Water movement across the CPE is *via *aquaporin 1, the waater channel which is abundantly expressed in the apical membrane and less so in the basolateral membrane (Figure 5B). This water channel is typical for epithelia that have a high rate of water transfer generated by a small osmotic gradient. Second, CSF to basolateral movement of potassium takes place [249,250]. However, it should be remembered that there are net fluxes of other ions across the CPE: Ca^2+^, organic anions and cations.

Although it would be possible for Na^+ ^to diffuse through paracellular spaces in the CPE (taking into account that TJs in the CP epithelium are relatively "leaky" due to the expression of certain claudins), analysis of electrochemical relations between the plasma and the CSF reveals that a gradient created by a CSF lumen-positive transepithelial voltage, which impedes Na^+ ^diffusion towards the CSF, exceeds the blood-to-ventricle chemical gradient for Na^+^; thus, the net movement of Na^+ ^from the blood side to the ventricle has to be an active ATP-dependent process [250]. Ion transporters expressed on the basolateral side of the CP epithelium load cells with Na^+^, while ion transporters expressed at the apical (CSF) side transport Na^+ ^from cells into the CSF. Two major anions, Cl^- ^and HCO_3_^- ^also pass the CPE layer *via *a transcellular route; the CSF concentrations of both Cl^- ^and HCO_3_^- ^are less than predicted for simple diffusion, which suggests that the paracellular route contributes negligibly to the overall transfer.

It is believed that the main entry route for Na^+ ^at the basolateral membrane is a stilbene-sensitive Na^+^- HCO_3_^- ^transporter (SLC4A10 or NBCn2/NBCE) (Figure 5B). This transporter was also known as Na^+^- dependent Cl^-^/HCO_3_^- ^exchanger, but the reported dependence of this transporter on intracellular Cl- has been disputed and the transporter was characterized as an electroneutral Na^+ ^- HCO_3_^- ^cotransporter [251]. In the CP, this transporter is expressed only in the basolateral membrane and transports Na^+ ^and HCO_3_^- ^into the cell [251]. Mice that had this transporter genetically removed had severe reduction in brain ventricle size, which suggested that the rate of CSF secretion was decreased [252]. This transporter is also important for loading CPE cells with HCO_3_^-^. The Na^+^, HCO_3_- cotransporter 1 (SLC4A7 or NBCn1) is also expressed in the CPE basolateral membrane [251] (Figure 5B), but evidence suggests that it does not play a major role in basolateral Na^+ ^loading and vectorial flux of this ion [250]. Cl^- ^accumulates in the CPE by action of the Cl^-^, HCO_3_^- ^-exchanger (anion exchange protein 2, SLC4A2 or Ae2) [253]. In the CPE, Cl^- ^movement intracellularly is driven by an outwardly-directed transmembrane gradient for HCO_3_^- ^that is created by the basolateral Na^+^, HCO_3_^- ^-cotransport [250].

Transport of these ions through the apical membrane to the CSF involves several proteins. Na^+^, K^+^- ATPase, which in the CPE is located exclusively in the apical membrane, is probably the most important Na^+ ^extruder [253] (Figure 5B). Three Na^+^, K^+^-ATPase subunits are expressed in CP epithelium at the transcript level: α1 (ATP1A1), β1 (ATP1B1) and β2 (ATP1B2) [240]. The Na^+^, H^+^-exchanger 1 (SLC9A1 or Nhe1) has also a predominantly apical localization in the CPE [212]; however, it is involved in intracellular pH regulation rather than in extrusion of Na^+ ^and it has been shown that Na^+^, H^+^-exchanger 1 knockout mouse lacked Na^+^-dependent intracellular pH recovery following acidification of CPE cells [254].

Transport of Cl^- ^across apical side is driven by a gradient towards the CSF and takes place mainly *via *K^+^-Cl^- ^-cotransporter 4 (SLC12A7 or KCC4), that is expressed on the apical side (Figure 5B). The apical side also expresses the Na^+^-K^+^-2Cl^-^- cotransporter 1 (SLC12A2 or NKCC1) [255], but its contribution to overall ion flux in this tissue is controversial because the net driving force for this transporter is close to zero due to high intracellular Na^+ ^and Cl- and low K^+ ^in the CSF [255], A recent report suggested that this transporter does not contribute to net ion fluxes [256]. Bicarbonate transport to the CSF across the apical surface is mediated mainly by the electrogenic Na^+^, HCO_3_^- ^-exchanger (SLC4A5 or NBCe2/NBC4) that couples transport of 1 Na^+ ^with 3 HCO_3_^- ^and has an important role in CSF pH regulation [244]. In amphibian CP HCO_3_^- ^flux across the apical membrane is mainly *via *Cl^- ^ion channel that has high permeability for HCO_3_^- ^[6]. This channel (Clir) plays some role in apical HCO_3_^- ^extrusion in mammals, but permeability of this channel for HCO_3_^- ^appears to be small.

It should be stressed that this section has focused only on molecular biology of transport for the principal ions: Na^+^, K^+^, Cl^- ^and HCO_3_^- ^and that it has not covered the transport of polyvalent ions such as Ca^++^, Mg^++^, PO_4_^3- ^and Fe^++^.

## Conclusion

Studies of the past two decades have provided insight into the molecular biology which underlines function of the two most important blood-brain fluid interfaces, the BBB and the BCSFB. Efficient homeostatic mechanisms established by those two barriers control composition of brain extracellular fluids, the ISF and CSF. These are vital to normal neuronal function and signal processing in the CNS. Two obvious functions that are common to the BBB and the BCSFB are the restriction of free diffusion and the transport of nutrients, waste products, signalling molecules and ions between blood and brain extracellular fluids. However, those two structures show important differences in their respective roles that are underlined by differences in expression of cell junction proteins, transport proteins and ion channels. An important similarity between the two barriers is that they are both dynamic systems and are able to respond rapidly to changes in brain requirements. The molecular basis of this feature is that the BBB and the BCSFB could be regulated *via *a number of molecular mechanisms under normal physiological or pathological conditions. Further insights into molecular mechanisms involved in BBB and BCSFB regulation should provide molecular cues for targeting the brain barriers in CNS diseases.

## List of abbreviations

α 2M: α2-macroglobulin; AA: amino acid; AD: Alzheimer's disease; AJ: adherens junctions; ANP: atrial natriuretic peptide; AVP: arginine vasopressin; BCRP: breast cancer resistance protein; BCSFB: blood-cerebrospinal fluid barrier; BDNF: brain-derived neurotrophic factor; BEC: brain endothelial cells; CAR: androstene receptor; CBF: cerebral blood flow; CP: choroid plexus; CPE: choroid plexus epithelium; CSF cerebrospinal fluid; DEP: diesel exhaust particles; DSIP: delta-sleep inducing peptide; EAAT: excitatory amino acid transporter; GDNF: glial cell line-derived neurotrophic factor; HIF-1: hypoxia-inducible factor 1; HUGO: Human Genome Organization; ISF: interstitial fluid; JAM: junctional adhesion molecules; JNK c-Jun N-terminal kinase; LAT: system-L amino acid transporter; LRP1: low-density lipoprotein receptor related protein-1; MCT: monocarboxylate transporter; MRP: multidrug resistance-related protein; NFκB: nuclear factor-κB; NGF: nerve growth factor; OAT: organic anion transporter; OATP: organic anion transporting polypeptide; P-gp: P-glycoprotein; PKC: protein kinase C; PTR: peptide transporters; PXR: pregnane-X receptor; qPCR- real time PCR; RAGE: receptor for advanced glycation end products; RAP: receptor-associated protein; SLC: solute carriers; TBI: traumatic brain injury; TEER: transendothelial/transepithelial electrical resistance; TGF: transforming growth factor; TJ: tight junction; TTR: transthyretin; VEGF: vascular endothelial growth factor; ZO: zonulla occludens;

## Competing interests

The authors declare that they have no competing interests.

## Authors' contributions

ZR: sole author. The author has read and approved the final version of the manuscript.
